# Modulation of the Autophagy-Lysosomal Pathway in Hepatocellular Carcinoma Using Small Molecules

**DOI:** 10.3390/molecules25071580

**Published:** 2020-03-30

**Authors:** Yu Geon Lee, Tae–Il Jeon

**Affiliations:** 1 School of Life Sciences, Ulsan National Institute of Science and Technology (UNIST), Ulsan 44919, Korea; ugun2@naver.com; 2 Department of Animal Science, Chonnam National University, Gwangju 61186, Korea

**Keywords:** autophagy, hepatocellular carcinoma, small molecules

## Abstract

Hepatocellular carcinoma (HCC) accounts for approximately 90% of all cases of primary liver cancer; it is the third most frequent cause of cancer-related death worldwide. In early-stage disease, surgical resection and liver transplantation are considered curative treatments. However, the majority of HCC patients present with advanced-stage disease that is treated using palliative systemic therapy. Since HCC is heterogeneous owing to its multiple etiologies, various risk factors, and inherent resistance to chemotherapy, the development of an effective systemic treatment strategy for HCC remains a considerable challenge. Autophagy is a lysosome-dependent catabolic degradation pathway that is essential for maintaining cellular energy homeostasis. Autophagy dysfunction is closely linked with the pathogenesis of various cancers; therefore, the discovery of small molecules that can modulate autophagy has attracted considerable interest in the development of a systemic treatment strategy for advanced HCC. Here, we reviewed the roles of autophagy in HCC and the recent advances regarding small molecules that target autophagy regulatory mechanisms.

## 1. Introduction

Hepatocellular carcinoma (HCC) is a malignant tumor with a high recurrence rate and poor prognosis. It is the third leading cause of cancer-related death worldwide [1]. The main risk factor contributing to HCC is liver cirrhosis, which predominantly occurs because of chronic hepatitis B (HBV) or C (HCV) infection. An emerging cause of HCC is non-alcoholic fatty liver disease (NAFLD), including non-alcoholic steatohepatitis (NASH) and fibrosis [2]. In fact, individuals with advanced fibrosis have shown a seven-fold higher risk of developing HCC than those without fibrosis. NAFLD-associated HCC results in a high mortality burden, indicating that NAFLD is emerging as a leading cause of HCC [3,4]. Currently, surgical resection, transplantation, and ablation are the most effective therapeutic strategies for patients with early-stage HCC. However, most HCC patients are not eligible for surgery at the time of diagnosis. Systemic therapy (with sorafenib, a multityrosine kinase inhibitor) is a critical therapeutic strategy for patients with advanced-stage HCC, but the overall median survival is less than 3 months [5]. Currently, other tyrosine kinase inhibitors including lenvatinib, regorafenib, and cabozantinib have been authorized as chemotherapeutic options for HCC treatment [6,7,8]. However, additional clinical trials are still required to confirm the expectation of their better clinical outcomes. Therefore, there is an unmet need for more efficient therapeutic strategies for HCC.

Autophagy is a highly conserved lysosome-dependent intracellular degradation process. It plays a critical role in maintaining cellular homeostasis [9]. According to the mechanism of cargo delivery to lysosomes, autophagy is classified into chaperone-mediated autophagy, microautophagy, and macroautophagy [10]. Among these, macroautophagy (hereafter referred to as autophagy) is well-studied. It is characterized by the involvement of double-membranous autophagosomes in the engulfment of intracellular components such as proteins and organelles. Autophagy occurs constitutively at a low level under normal conditions; it is rapidly upregulated by internal or external stresses such as oxidative injury, protein aggregation, organelle damage, and attack by pathogens [11]. Emerging evidence indicates that autophagy dysregulation is linked to the pathogenesis of liver diseases such as NASH, fibrosis, cirrhosis, and HCC [12]. Autophagy can suppress initial and persistent liver injury, preventing its progression to liver cancer. However, increased autophagy often enables tumor cell survival and growth, suggesting that autophagy could serve as a hepatoprotective mechanism as well as a facilitator of tumorigenesis, tumor evasion, and chemoresistance in HCC [13]. Considering the importance of these pathological mechanisms, autophagy has gained intense interest as a promising target for the development of therapies against HCC.

Therefore, here, we review recent advances regarding the discovery of small molecules that can target the various regulatory mechanisms of autophagic machinery and highlight the potential therapeutic applications of these compounds in HCC.

## 2. Mechanisms of Autophagy

**Figure 1:**
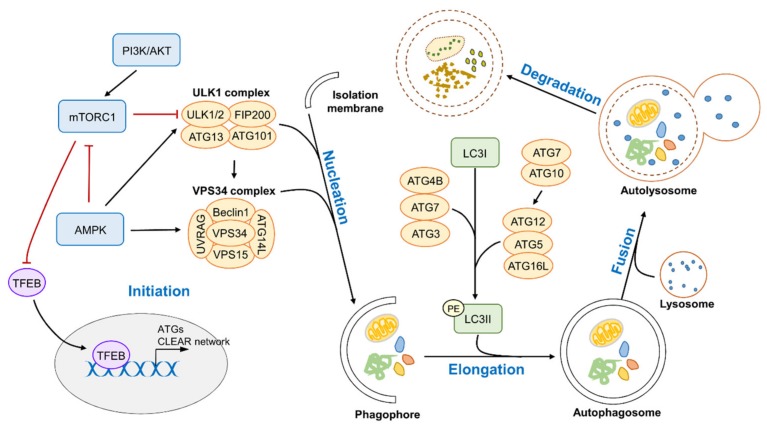
The main pathway for autophagy modulation. The several groups of autophagy-related genes including ULK1 complex, VPS34 complex, and ATG12-ATG5-ATG16L complex involved in autophagosome formation. Depletion of growth factor and/or nutrient cause the inactivation of mammalian target of rapamycin complex 1 (mTORC1) that acts as an autophagy suppressor by inhibiting ULK1 genes. AMP-activated protein kinase (AMPK) positively modulates autophagy machinery via inhibition of mTORC1 and activation of either ULK1 complex or VPS34 complex. ER-stress mediated unfolded protein response (UPR) stimulates AMPK-driven autophagy and is also involved in the process for autophagosome formation (not shown). Activated ULK1 complex resulted in activating VPS34 complex, facilitating the autophagy process including nucleation and elongation. Conversion of LC3I into phosphatidylethanolamine (PE)-LC3II, a key substrate for autophagosome membrane assembly, is mediated by two ubiquitin-like conjugation systems including ATG4B, ATG7, ATG3, and ATG12-ATG5-ATG16L complex. Subsequently, closed autophagosome fuses with lysosome to form an autolysosome, which leads to autophagy degradation of cytoplasmic components. This process is sustained by preventing the mTORC1-mediated phosphorylation of transcription factor EB (TFEB), which induces the expression of genes involved in autophagosome and lysosome biogenesis. CLEAR, coordinated lysosomal expression and regulation motif; PI3K, phosphoinositide-3-kinase; AKT, protein kinase B; UVRAG, UV radiation resistance-associated gene protein.

A series of protein complexes composed of autophagy-related gene (ATG) products coordinates the autophagy process that consists of initiation, nucleation, elongation, maturation, fusion, and degradation (Figure 1). Initiation of the autophagy process is governed by the ULK1 (also known as ATG1) complex (ULK1, FIP200, ATG101, and ATG13) that triggers phagophore formation. Under starvation or other stress conditions, the ULK1 complex is activated by the inactivation of mammalian target of rapamycin complex 1 (mTORC1), which lies downstream of phosphoinositide-3-kinase (PI3K)/protein kinase B (AKT) [14]. mTORC1 can be inhibited by AMP-activated protein kinase (AMPK), which also directly catalyzes the activating phosphorylation of ULK1 and beclin 1 [15,16]. Subsequently, the activated ULK1 complex is redistributed on the endoplasmic reticulum (ER) surface and recruits the class III PI3K (PI3KC3) complex (VPS34, beclin 1, and VPS15), leading to the synthesis and deposition of phosphatidylinositol-3-phosphate (PI3P) at the autophagosome elongation site. This step further facilitates the recruitment of PI3P-binding proteins, such as WD repeat domain phosphoinositide-interacting protein (WIPI2) and zinc-finger FYVE domain-containing protein 1 (DFCP1), resulting in the localization of Atg proteins on the autophagosomal membrane [17]. The PI3KC3 complex interacts with a variety of additional regulatory factors, including ATG14L, UV radiation resistance-associated gene protein (UVRAG), and autophagy and beclin 1 regulator 1 (AMBRA1), promoting phagophore formation (Figure 1) [18,19].

There are two ubiquitin-like conjugation systems required for phagophore elongation. The first system is mediated by the E1-like enzyme ATG7 and E2-like enzyme ATG10, leading to the formation of the ATG12-ATG5-ATG16L1 complex, which acts as an E3-like enzyme. The second junction system involves the proteases ATG4B, ATG3, and ATG7 and promotes the conjugation of phosphatidylethanolamine (PE) to cytoplasmic microtubule-associated protein 1 light-chain 3 (LC3I) to generate its lipidated form LC3II, which is incorporated into the growing membrane. The completed autophagosome fuses with a lysosome to form an autolysosome. The low pH of the lysosome allows the degradation of the autophagic cargoes in the lysosomal compartment, thereby supplying energy and substrates to maintain cell homeostasis under stress conditions (Figure 1) [20].

In addition to post-translational modifications of autophagy-related proteins, transcriptional mechanisms are also necessary for a sustained response. The transcriptional network that controls autophagy is complex and involves a number of transcription factors. Among these, transcription factor EB (TFEB) and the forkhead box O (FOXO) have been shown to be the key transcriptional regulators of autophagy and lysosomal biogenesis. TFEB is a member of the microphthalmia/transcription factor E (MiT/TFE) family that also includes MITF, TFE3, and TFEC proteins [21]. Under nutrient-rich conditions, mTORC1 is sufficient to induce phosphorylation of TFEB and sequester it into the cytosol, inhibiting the expression of lysosomal/autophagic genes including ATG4, ATF9B, SQSTM1, WIPI, and UVRAG [22]. Conversely, under starvation or stress conditions, TFEB is dephosphorylated and translocated into the nucleus, subsequently promoting the expression of target genes involved in diverse steps of the autophagy process, including lysosome biogenesis [23]. Similar to TFEB, FOXOs are regulated by nuclear translocation via phosphorylation to induce the expression of autophagy-related genes, including ATG4, ATG5, ATG12, ATG14, BECN1, BNIP3, ULK1, and VPS34 [24]. AKT, stimulated by growth factors or insulin, mostly mediates the phosphorylation of FOXOs, inhibiting the transcriptional activation of these genes [25].

## 3. Role of Autophagy in HCC

Since autophagic pathways contribute to homeostasis preservation and stress adaptation in normal hepatocytes, they can suppress the tumorigenic process in the liver. Indeed, autophagy deficiency, due to liver-specific deletion of ATG7 or ATG5, promoted spontaneous liver tumorigenesis, the accumulation of damaged mitochondria and sequestome-1 (SQSTM1/p62), oxidative stress, and genome damage responses in mice [26,27]. Aggregation of p62 sequesters kelch-like ECH-associated protein 1 (KEAP1), a component of the ubiquitin ligase complex for nuclear factor erythroid 2-related factor 2 (NRF2), and causes inappropriate activation of NRF2 target genes, including those encoding antioxidant proteins and hepatic detoxification enzymes [28,29]. Interestingly, deletion of p62 or NRF2 in mice with liver-specific ATG7 or ATG5 deficiency decreased liver tumor development, implying that the p62-KEAP1-NRF2 axis contributes to tumorigenesis [26,30]. Moreover, p62 accumulation and NRF2 activation were found in HCC patients and were correlated with a poor overall survival rate in HCC caused by HBV and aflatoxin B1 [31,32]. Thus, the p62-KEAP1-NRF2 axis could serve as the key driver of human HCC. Human antigen R (HuR), a member of the Hu/ELAV-like RNA binding protein family, can post-transcriptionally regulate p62 [33], and depletion of HuR in HCC cell lines resulted in a decrease of both mRNA and protein expression of ATG5, ATG12, and ATG16 [34]. In addition, ATG5 and ATG7 expressions from HCC tissue were positively correlated with HuR expression, suggesting that HuR has tumorigenic capacity during autophagy-driven HCC progression. Autophagy defects in liver-specific ATG7 knockout mice also resulted in the activation of Yap, a nuclear effector of the Hippo signaling pathway, which is an early event in liver cancer development. Yap deletion in such mice attenuated hepatocarcinogenesis despite a preserved p62-NRF2 axis, implying that Yap is an independent driver of HCC in autophagy-impaired livers [35].

Beclin 1 was found to be mono-allelically deleted in over 50% of patients with breast and ovarian cancers and expressed at reduced levels [36]. Heterozygous disruption of beclin1 in mice reduced autophagy, thereby increasing spontaneous HCC tumorigenesis and facilitating the progression of the HBV-induced premalignant phenotype [37,38]. In addition, beclin 1 expression was lower in HCC tissues than in non-cancerous tissues and positively correlated with the 5-year survival rate of HCC patients [39,40,41], suggesting that beclin 1-mediated autophagy during HCC progression acts as a tumor-suppressing mechanism.

Ubiquitination of UVRAG by SMURF1, homologous to E6AP C-Terminus (HECT)-type ubiquitin ligase, can cause autophagy vesicle maturation and subsequent decrease of epidermal growth factor receptor signaling and inhibition of HCC tumor growth [42]. Intriguingly, binding of SMURF1 to UVRAG can be dissociated by UVRAG phosphorylation, resulting in disruption of UVRAG ubiquitination-mediated autophagosome maturation. Indeed, increase of UVRAG phosphorylation was correlated with poor outcomes of HCC patients, implying that each type of post-translational modification of ATGs including UVRAG might have a different function in HCC progression [42].

However, autophagy also promotes tumor progression and therapeutic resistance in cancer patients [43]. In fact, starvation-induced autophagy promotes HCC cell invasion via the activation of epithelial-mesenchymal transition involving TGF-β/SMAD3 signaling [44]. In addition, the stable depletion of beclin 1 and ATG5 significantly reduced the lung metastasis of HCC cells that were subcutaneously or orthotopically xenografted into nude mice by impairing anoikis-resistance and lung colonization, suggesting that autophagy promotes HCC metastasis [45]. In another study, miR-375 was downregulated in human HCC tissues. Moreover, the stable expression of miR-375 inhibited autophagy by targeting ATG7, resulting in the reduction of HCC cell viability under hypoxic and tumor growth conditions in mice [46]. These results also suggested that autophagy could support HCC cell survival in a hypoxic or low-nutrient environment, thus promoting HCC malignancy. Clinical studies also demonstrated that LC3II, a marker of autophagosome formation, was positively correlated with metastasis and poor prognosis in HCC patients [47,48]. However, since either increased autophagic flux or a block in autophagic flux can result in high LC3II levels, further investigations are necessary to assess the causal association between autophagic flux and increased LC3II in HCC. Furthermore, autophagy induction in response to chemotherapy contributes to chemoresistance and apoptosis-resistance in HCC [49,50]. For example, chemical inhibitors of autophagy, such as chloroquine and 3-methyladenine, improved chemotherapy or radiotherapy in a xenograft mouse model of liver cancer, suggesting that autophagy serves as a cytoprotective mechanism in stress conditions [51,52].

Collectively, the role of autophagy in HCC can be altered depending on the stage of HCC development. Autophagy can support HCC proliferation in the dysplastic stage by enhancing HCC survival to sustain its metastatic potential, whereas it can also exert a tumor-suppressive effect in the tumor-forming stage by removing toxic components that affect tumor initiation. However, the molecular mechanism that fine-tunes this dynamic process remains unclear.

## 4. Autophagy Modulation in HCC by Small Molecules

Research to date indicates that autophagy modulators could be used as pharmacological agents to overcome the limitations of chemotherapy, such as drug toxicity and resistance, in HCC treatment [53]. In this context, several small molecules have emerged as potential anti-HCC drugs that target the autophagy pathway. In the following section, we discuss how small molecules modulate autophagy and HCC cell death or survival, and summarize their effects in HCC (Table 1).

### 4.1. Cytoprotective Autophagy by Small Molecules

Autophagy inhibition by small molecules is a promising therapeutic strategy for HCC because autophagy allows cells to survive and grow under stress conditions such as chemotherapy. Indeed, single treatment with another lysosome inhibitor, bafilomycin A1 or chloroquine, significantly suppressed HCC tumor growth in a xenograft model by impairing autophagic flux and arresting the cell cycle [55,73]. In addition, several phase I/II clinical studies supported the idea that inhibiting autophagy using an inhibitor of lysosomal degradation, chloroquine or hydroxychloquine, in combination with conventional anticancer treatments, could improve clinical outcomes for HCC patients without causing serious adverse events [91]. In preclinical studies, multikinase inhibitors sorafenib and linifanib induced the activation of autophagic flux, suppression of the mTOR and mitogen-activated protein kinase kinase (MEK)/extracellular signal-regulated kinases (ERK) signaling pathways in HCC cells, and pharmacological inhibition of autophagy enhanced cell death. Moreover, combination therapy, using chloroquine, hydroxychloquine, or verteporfin (FDA-approved photosensitizer) with sorafenib and linifanib, resulted in more pronounced HCC tumor suppression than single treatment with these drugs in a mouse xenograft model and in HCC patient–derived tumoroids [65,66,68], suggesting that blockage of autophagy could be a promising strategy to overcome chemoresistance.

Although platinum-based anticancer drugs oxaliplatin or cisplatin have been widely used in the treatment of various cancers, these drugs showed modest and limited efficacy against advanced HCC in clinical trials [92]. Recent studies demonstrated that oxaliplatin or cisplatin treatment induced cytoprotective autophagy in HCC cells and xenografts. Moreover, inhibition of autophagosome formation or lysosomal degradation using 3-methyladenine or chloroquine enhanced the sensitivity to monotherapy with these drugs via an increase in the level of ROS [54,61]. Similarly, the protein kinase C (PKC) inhibitor bisindolylmaleimide alkaloid 155Cl (BMA-155Cl) induced autophagy, which was associated with the NF-kB p65 signaling pathway, in HepG2 cells and xenografts to evade apoptosis [93]. Notably, the chemical inhibition of NF-κB p65 by BAY 11-7082 suppressed BMA-155Cl-induced autophagy and apoptosis via downregulation of the autophagy protein LC3B and proapoptotic proteins, indicating the dual regulatory function of NF-κB in autophagy and apoptosis.

Since the mTOR signaling pathway is hyperactivated in 40%–50% of HCC cases and associated with poor prognosis, genetic and pharmacological inhibition of the mTOR pathway has emerged as an attractive therapeutic strategy for HCC. Indeed, a number of mTOR inhibitors are currently under evaluation in preclinical or clinical trials for HCC. However, the efficacy of the various types of mTOR inhibitors, including first- and second-generation drugs, is limited in HCC therapy owing to drug resistance and compensatory activation of other signaling pathways [94]. Although the molecular mechanisms of the mTOR pathway in chemoresistance have not been well established, one possible reason for the limited efficacy of these inhibitors is that they activate cytoprotective autophagy, contributing to drug resistance involving various signaling pathways. For instance, the potential mTOR kinase inhibitor KU-0063794 induced significant apoptotic and autophagic responses in HepG2 cells, however, autophagy inhibitors dramatically enhanced its cytotoxic activity. Moreover, the antitumor activity of KU-0063794 in the mouse xenograft model of HCC was further enhanced by co-administration of 3-methyladenine, indicating that autophagy is a factor responsible for chemoresistance to mTOR kinase inhibitor treatment [95]. Over the past decade, accumulating lines of evidence have suggested that cancer stem-like cells (CSCs) are responsible for chemoresistance, tumor relapse, and metastasis owing to their capacity to self-renew and differentiate into the heterogeneous lineages of cancer cells in response to chemotherapeutic agents [96]. It has been reported that the presence of CSCs is associated with the poor survival outcome of advanced HCC patients. Further, the expression of CSC’s marker, CD133, is correlated with this clinical outcome [97], suggesting that the subpopulation of CD133^+^ liver CSCs plays a critical role in tumorigenesis and chemoresistance. A recent study showed that a treatment with a rapamycin analog inhibitor of mTOR, temsirolimus (CCI779), significantly induced CD133 expression in liver CSCs and human HCC cells, and combination treatment with a flavonoid baicalein and temsirolimus eliminated chemoresistance to mTOR inhibition in liver CSCs and the patient-derived xenograft model of HCC. Mechanistically, baicalein blocks the autophagy induced by temsirolimus by interfering with the GTP binding of SAR1B GTPase, which is essential for autophagy activation [74]. Similar to baicalein, epigallocatechin-3-*O*-gallate (EGCG), the most abundant catechin in green tea, suppressed autophagosome formation and proliferation in HCC cells. Treatment with EGCG synergistically promoted the induction of apoptosis by doxorubicin, an anticancer agent used for HCC, and the reduction of autophagic flux, thereby inhibiting the growth of Hep3B xenograft tumors [57].

It has been reported that most natural product-derived small molecules induce cytoprotective autophagy via diverse mechanisms in HCC in response to apoptosis for survival. A BCL-2 inhibitor and gossypol derivative, apogossypolone [71], and capsaicin [77] induced apoptotic cell death and autophagy by either disrupting the interaction between beclin 1 and BCL-2/BCL-XL or increasing ROS generation. Notably, autophagy inhibition by antioxidants enhanced the chemosensitivity of HCC cells to apogossypolone and capsaicin. Similarly, it was reported that gartanin, an isoprenylated xanthone, induced both autophagy and apoptosis in HCC cells. Moreover, 3-methyadenine treatment resulted in the further suppression of HCC growth. Autophagy induction by gartanin was selectively mediated by c-Jun N-terminal kinase (JNK) activation and subsequent phosphorylation of BCL-2, ultimately leading to beclin 1 activation [80]. Arenobufagin, a bufadienolide [72], and matrine [82,83], a main alkaloid component from Sophora root, induced mitochondria-mediated apoptosis by repressing the PI3K/AKT/mTOR pathway in HepG2 cells and autophagy inhibition enhanced apoptotic cell death, indicating that arenobufagin-induced autophagy protects from undergoing cells. An ATP-competitive dual PI3K/mTORC1/C2 inhibitor and an imidazoquinoline derivative, NVP-BGT226, also induced G_0_/G_1_ cell cycle arrest, apoptosis, and cytoprotective autophagy in various HCC cell lines [84]. Gallotannin, a hydrolysable tannic acid derivative, induced caspase-dependent apoptosis and autophagy by repressing SIRT1 and activating the AMPK signaling pathway in HepG2 and SK-Hep1 cells, and a xenograft tumor model and the chemical inhibition of autophagy flux enhanced the antitumor effects of gallotannin [79]. Platycodin D, a triterpenoid saponin, exerted anti-HCC effect by autophagy activation through the MEK/ERK and JNK signaling pathways and can help HCC cells escape from apoptotic cell death [86,87,88]. Although the precise mechanism for the control of autophagy by apoptosis and tumor suppressor genes requires further investigation, inhibition of autophagy by autophagy inhibitors (alone or in combination with small molecules that induce the survival mechanism of autophagy) can be used as an effective therapeutic strategy for HCC.

Autophagy can help cells cope with ER stress, such as the accumulation of unfolded or aggregated proteins, or participate in the mechanism of ER stress-induced cell death [98]. Li et al. [99] found out that xanthoangelol, a natural prenylated chalcone, markedly suppressed the growth of HCC cells and SMMC 7721 tumor xenografts. Moreover, xanthoangelol treatment induced apoptosis and protective autophagy, mediated by the stimulation of ER stress via the activation of the c-Jun N-terminal kinase (JNK)/c-jun axis. Bufalin (a cardiotonic steroid) [76] and anthocyanins [70] also induced ER stress-mediated protective autophagy via ER stress sensors, the inositol-requiring enzyme 1 (IRE1)/JNK and protein kinase RNA-like ER kinase (PERK)/eukaryotic initiation factor 2α (eIF2α) signaling pathways, respectively, leading to the apoptosis of HCC cells. These studies indicated that the activation of autophagy by chemotherapy reagents after ER stress was involved in the development of drug resistance to HCC, despite the fact that complex linkages among ER stress, autophagy, and apoptosis remain unclear.

### 4.2. Autophagic Cell Death by Small Molecules

Emerging evidence has revealed that abnormal or excessive autophagy is sufficient to induce non-apoptotic programmed cell death (PCD), also known as autophagic cell death or type II PCD [100,101]. Autophagic cell death can be distinguished from apoptosis (type I PCD) or necrosis (type III PCD) and is characterized by morphological changes as well as the activation of specific signaling pathways [102,103]. Autophagy modulation is essential for cell death under certain circumstances, especially those that involve defective apoptosis and resistance to apoptosis-targeting chemotherapy [104]. Moreover, the activation of additional signaling pathways is required for autophagy-induced cell death, suggesting that understanding the alterations in the various cell-death-signaling pathways mediated by autophagy modulation is important for the development of therapeutic agents that can target autophagy in HCC [105].

Sorafenib, a multiprotein kinase inhibitor, is a drug that has demonstrated survival advantages in patients with advanced HCC [106]. Although several lines of evidence have established that sorafenib induces a protective form of autophagy for HCC cell survival, a recent study demonstrated that sorafenib and its kinase-independent derivative, SC-59, induced autophagic cell death and apoptosis by disrupting the myeloid cell leukemia-1 (MCL-1)-beclin 1 complex in HCC cells and PLC5 xenograft nude mice [64]. Interestingly, the combination treatment of sorafenib with antifolate drug pemetrexed synergistically enhanced autophagy in Huh7 cells, inducing cell death, and the inhibition of autophagy by beclin 1 knockdown suppressed the cytotoxic interaction between sorafenib and pemetrexed [63]. On the contrary, pemetrexed also induced MEK/ERK-mediated cytoprotective autophagy in HepG2 cells, suggesting autophagy activation by a p53-dependent or -independent mechanism in HCC [62].

In addition, a derivative of celecoxib, a cyclooxygenase-2 inhibitor known as OSU-03012, inhibited the growth of HCC cells and Huh7 tumor xenografts via reactive oxygen species (ROS)- induced autophagy without affecting apoptosis. These effects were blocked by 3-methyladenine, suggesting that OSU-03012 induces autophagic cell death in HCC [85]. Nilotinib, a second-generation tyrosinase kinase inhibitor used for treating leukemia, and AZD8055, an inhibitor of mTORC1 and mTORC2, also induced autophagic cell death in HCC cells by activating AMPK signaling but showed no effect on apoptosis [60,69]. Dihydroartemisinin (DHA), an FDA-approved antimalarial drug and a potent inhibitor of mTORC1, promoted AIM2/caspase-1 inflammasome and ROS production, which contribute to autophagy-mediated cell death in HCC cells [56]. Notably, combination treatment with rapamycin and metformin, as an AMPK activator, can further enhance autophagic cell death in the mouse xenograft model of HCC, reducing drug resistance with mTOR inhibitor [59]

In addition to small molecule drugs, several natural-product-derived small molecules have been reported to exert anti-proliferative effects in HCC by targeting autophagy. Recently, we investigated the effects of an urushiol derivative 3-decylcatechol (DC) on Huh7 cells. We found that DC promoted autophagic cell death and necrosis; moreover, the stimulation of ER stress by DC induced autophagy via p62 transcriptional activation involving an ER stress sensor, the IRE1/JNK pathway [67]. Similarly, dehydroepiandrosterone increased p62 transcription via activation of the JNK-NRF2 axis, thus promoting autophagic cell death in HepG2 cells [78]. Pterostilbene, a natural dimethylated analog of resveratrol, inhibited the proliferation of HCC cells and growth of SK-Hep-1 xenograft tumors via ER stress-mediated autophagy activation. Inhibition of autophagy by 3-methyladenine resulted in decreased vacuolization, which was increased by pterostilbene. The PERK/eIF2α/activating transcription factor-4 (ATF4) axis was responsible for the induction of autophagic cell death, without apoptosis, by pterostilbene [89]. These studies suggest a mechanistic link between the unfolded protein response (UPR) signaling pathway that is triggered by ER stress and autophagy. In fact, UPR transducers (IRE1, PERK, and ATF6) regulate autophagy in different ways during ER stress. For example, IRE1 activation leads to the dissociation of BCL-2 from beclin 1 via JNK phosphorylation, thereby activating beclin 1 and the PI3K complex, which stimulate autophagy [107,108]. In addition to kinase activity, endonuclease activity of IRE1 induces splicing of XBP1, resulting in transcriptional upregulation of beclin 1 to sustain autophagic flux. The PERK/ATF4 pathway of UPR also increases the transcription of MAP1LC3, beclin 1, ATG3, ATG12, ATG5, and other autophagy genes. These lines of evidence indicate that ER stress-mediated autophagy is an important target in HCC therapy. However, ER stress-induced autophagy can also act as a cytoprotective mechanism [58,98]. Thus, it is important to understand the molecular interaction and functional relationship between UPR and autophagy in HCC.

Flavonoids have recently been found to exert anticancer properties through various cellular processes, including autophagy. Quercetin was found to induce autophagy-mediated apoptosis via mitogen-activated protein kinase (MAPK) and the AKT/mTOR pathway, resulting in growth inhibition of SMMC7221 tumors [90]. It was also reported that luteolin, a flavone type of flavonoid, exerted antitumor effects via autophagy, cell cycle arrest, and apoptosis in SMMC-7721 cells [109]. A flavonol type of flavonoid, kaempferol, inhibited the proliferation of HCC cell lines and primary human HCC cells via the induction of autophagy and apoptosis by AMPK activation [81]. Kaempferol treatment resulted in the inactivation of mTORC1 signaling, which was restored by AMPK inhibition with either small hairpin RNA (shRNA) for AMPKα1 or dominant negative mutation. AMPK activation by kaempferol upregulated the ULK1 signaling pathway, thereby increasing autophagic flux. Either autophagy inhibition or AMPK inactivation protected HCC cells from kaempferol-induced cell death, indicating autophagic cell death.

Zhang et al. [110] presented evidence that resveratrol triggered autophagy and exerted antitumor effects in HCC cells. In addition, autophagy inhibition by 3-methyladenine can reduce the inhibitory effect of resveratrol on the metastatic capacity of HCC cells. Moreover, the decreased tumor growth mediated by resveratrol increased upon treatment with a p53 inhibitor. However, resveratrol induced autophagic cell death by the induction of autophagy-related genes through a p53-independent pathway [111], providing evidence of the interplay between autophagy machinery and tumor suppressor p53 genes. Wang et al. [75] reported that berberine, an isoquinoline plant alkaloid, exhibited cytotoxicity by certain mechanisms, including apoptosis and autophagy, in HepG2 and MHCC97-1 cells. They observed that berberine treatment induced autophagy by activating beclin 1 and inactivating the AKT/mTOR pathway. Further, genetic deletion of ATG5 resulted in decreased cytotoxic activity of berberine. These studies suggested that an intricate interplay between autophagy and other cell-death mechanisms, such as apoptosis, cell cycle arrest, and necrosis, contributes to the tumor-suppressing function of small molecules in HCC therapy.

## 5. Conclusions

**Figure 2:**
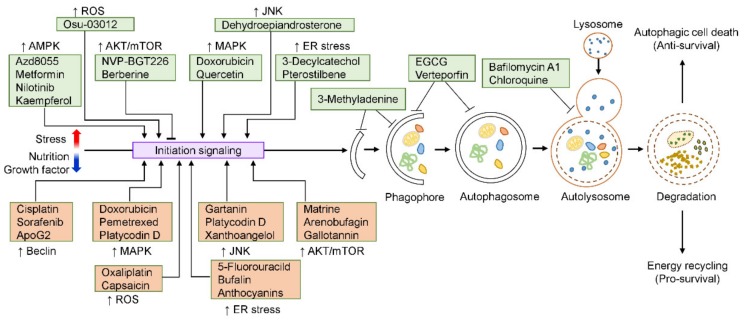
Representative autophagy-modulating small molecules in HCC. Diverse small molecules can modulate autophagy in HCC at different steps. Small molecules capable of activating (↑) or inhibiting (↓) autophagy through the regulation and autophagy-related signaling and process are proposed. Small molecules that affect HCC cell fate by autophagy modulation are shown in green boxes (cell death) and orange boxes (cell survival). ApoG2, Apogossypolone; EGCG, (-)-Epigallocatechin-3-*O*-gallate; ROS, reactive oxygen species; JNK, c-Jun N-terminal kinase; ER. endoplasmic reticulum; AKT/mTOR, protein kinase B/mammalian target of rapamycin; MAPK, mitogen-activated protein kinase.

Although using small molecules to target the autophagy regulatory mechanism is a promising therapeutic approach for HCC, limitations in the clinical application of autophagy modulators for HCC therapy exist and must be addressed. In particular, mistargeting of autophagy, which results in unfavorable outcomes owing to the multiple functions of autophagy and the non-autophagic roles of Atgs under different pathophysiological conditions, must be considered [112]. Thus, increasing tumor-selective autophagy using autophagic modulators or minimizing their cross-talk with other targets is required for their clinical application. Additionally, since autophagy plays a physiologically important role in normal cells, it must be clarified whether autophagy modulation adversely affects healthy cells. Natural small molecules have great benefits in the treatment of autophagy-related diseases, including cancer, owing to their low toxicity. Thus, the development of anti-HCC drugs using natural small molecules that target autophagy could be a good strategy (Figure 2).

## Figures and Tables

**Table 1:** Targeting autophagy using small molecules in HCC.

Name	Category	Molecular Mechanism	Autophagy Modulation	Role of Autophagy	Ref
Cisplatin	FDA-approved drug	Activates beclin 1	Inducer	Survival	[54]
Chloroquine	FDA-approved drug	Inhibits autophagosome–lysosome fusion	Inhibitor	Cell death	[55]
Dihydroartemisinin	FDA-approved drug	Promotes AIM2/caspase-1 inflammasome	Inducer	Cell death	[56]
Doxorubicin	FDA-approved drug	Activates MAPK signaling	Inducer	Survival	[57]
5-Fluorouracil	FDA-approved drug	Activates ER stress (CHOP/GADD153)	Inducer	Survival	[58]
Metformin	FDA-approved drug	Activates AMPK signaling	Inducer	Cell death	[59]
Nilotinib	FDA-approved drug	Activates AMPK signaling	Inducer	Cell death	[60]
Oxaliplatin	FDA-approved drug	Increases cellular ROS level	Inducer	Survival	[61]
Pemetrexed	FDA-approved drug	Increases beclin 1/Activates MAPK signaling	Inducer	Cell death/Survival	[62,63]
Sorafenib	FDA-approved drug	Activates beclin 1/Inhibits MAPK signaling	Inducer	Cell death/Survival	[64,65]
Verteporfin	FDA-approved drug	Decrease lysosomal membrane stability	Inhibitor	Cell death	[66]
3-Decylcatechol	Urushiol derivative	Activates inositol-requiring enzyme 1 (IRE1) /JNK signaling	Inducer	Cell death	[67]
3-Methyladenine	Purine derivative	Inhibits type III phosphoinositide-3-kinase (PI3K)	Inhibitor	Cell death	[68]
Azd8055	mTORC1/2 inhibitor	Activates AMPK signaling	Inducer	Cell death	[69]
Anthocyanins	Flavonoid	Activates IRE1/JNK signaling	Inducer	Survival	[70]
Apogossypolone	Gossypol derivative	Promotes dissociation of beclin-1 with Bcl-2	Inducer	Survival	[71]
Arenobufagin	Steroid	Inhibits AKT/mTOR signaling	Inducer	Survival	[72]
Bafilomycin A1	Macrolide antibiotic	Inhibits autophagosome-lysosome fusion	Inhibitor	Cell death	[73]
Baicalein	Flavonoid	Inhibits SAR1B GTPase	Inhibitor	Cell death	[74]
Berberine	Alkaloid	Inhibits AKT/mTOR signaling	Inducer	Cell death	[75]
Bufalin	Steroid	Activates IRE1/JNK signaling	Inducer	Survival	[76]
Capsaicin	Capsaicinoids	Increase STAT-dependent ROS generation	Inducer	Survival	[77]
Dehydroepiandrosterone	Steroid	Activates JNK-NRF2-p62 signaling	Inducer	Cell death	[78]
(−)-Epigallocatechin-3-*O* -gallate	Flavonoid	Inhibits autophagosome formation	Inhibitor	Cell death	[57]
Gallotannin	Tannin	Inhibits AKT/mTOR signaling	Inducer	Survival	[79]
Gartanin	Xanthone	Activates JNK signaling	Inducer	Survival	[80]
Kaempferol	Flavonoid	Activates AMPK signaling	Inducer	Cell death	[81]
Matrine	Alkaloid	Inhibits AKT/mTOR signaling	Inducer	Survival	[82,83]
NVP-BGT226	PI3K/mTOR inhibitor	Inhibits AKT/mTOR signaling	Inducer	Cell death	[84]
Osu-03012	celecoxib derivative	Increases cellular ROS level	Inducer	Cell death	[85]
Platycodin D	Saponin	Activates MAPK signaling Activates JNK signaling	Inducer	Survival	[86,87,88]
Pterostilbene	Stilbenoid	Activates PERK/eIF2α signaling	Inducer	Cell death	[89]
Quercetin	Flavonoid	Activates MAPK signaling	Inducer	Cell death	[90]
